# Monitoring Response and Resistance to the Novel Arsenical Darinaparsin in an AML Patient

**DOI:** 10.3389/fphar.2013.00009

**Published:** 2013-02-12

**Authors:** Torsten H. Nielsen, Nathalie Johnson, Nicolas Garnier, Stanley Kwan, Lu Yao, Eftihia Cocolakis, Josée Hébert, Robert A. Morgan, Éric Paquet, Kevin P. Callahan, Craig T. Jordan, Sarit Assouline, Wilson H. Miller, Koren K. Mann

**Affiliations:** ^1^Segal Cancer Center, Lady Davis Institute for Medical Research, McGill UniversityMontréal, QC, Canada; ^2^Leukemia Cell Bank of Québec, Maisonneuve-Rosemont HospitalMontréal, QC, Canada; ^3^Division of Hematology-Oncology, Maisonneuve-Rosemont HospitalMontréal, QC, Canada; ^4^ZIOPHARM Oncology Inc.New York, NY, USA; ^5^Department of Biochemistry, McGill UniversityMontréal, QC, Canada; ^6^University of Rochester Medical CenterRochester, NY, USA

**Keywords:** acute myeloid leukemia, inv(3)(q21q26.2), darinaparsin, experimental treatment, resistance, personalized medicine

## Abstract

Acute myeloid leukemia (AML) with inversion of chromosome 3 is characterized by overexpression of EVI1 and carries a dismal prognosis. Arsenic-containing compounds have been described to be efficacious in malignancies overexpressing EVI1. Here, we describe a case of AML with inv(3)(q21q26.2) treated with the organic arsenical darinaparsin. Using a “personalized medicine approach,” two different arsenicals were screened for anti-leukemic effect against the patient’s cells *ex vivo*. The most promising compound, darinaparsin, was selected for *in vivo* treatment. Clinical effect was almost immediate, with a normalization of temperature, a stabilization of white blood cell (WBC) counts and an increased quality of life. Longitudinal monitoring of patient response and resistance incorporating significant correlative studies on patient-derived blood samples over the two cycles of darinaparsin given to this patient allowed us to evaluate potential mechanisms of response and resistance. The anti-leukemic effects of darinaparsin correlated with inhibition of the alternative NF-κB pathway and production of the inflammatory cytokine IL-8. Emergence of resistance was suspected during treatment cycle 2 and supported by xenograft studies in nude mice. Darinaparsin resistance correlated with an attenuation of the effect of treatment on the alternative NF-κB pathway. The results from this patient indicate that darinaparsin may be a good treatment option for inv(3) AML and that inhibition of the alternative NF-κB pathway may be predictive of response. Longitudinal monitoring of disease response as well as several correlative parameters allowed for the generation of novel correlations and predictors of response to experimental therapy in a heavily pretreated patient.

## Introduction

Acute myeloid leukemia (AML) with inversion or translocation of the long arm of chromosome 3 (inv(3; q21q26.2)/t(3;3; q21;q26.2), hereafter referred to as “inv(3)/t(3;3) AML” accounts for 1–2% of AML cases and predicts an extremely poor prognosis, with a 5-year overall survival of 5.7% (Lugthart et al., 2010). Due to the distinct clinicopathologic attributes associated with these chromosomal aberrations, inv(3)/t(3;3) AML has been included as a separate sub-category of “AML with recurrent genetic abnormalities” in the latest WHO classification (Swerdlow et al., 2008). Despite a characteristic clinical picture with a well-defined underlying molecular pathology, no targeted therapy currently exists for inv(3)/t(3;3) AML, although improved treatment regimens clearly are needed, as reflected by the poor overall performance of this patient category.

Molecularly, inv(3)/t(3;3) AML is characterized by the aberrant juxtaposition of the oncogene ecotropic viral integration site 1 (EVI1), with regulatory elements of ribophorin 1 (RPN1), resulting in overexpression of EVI1 (Buonamici et al., 2003). EVI1 is a transcription factor with a well-recognized role in the normal development of the hematopoietic system. It has been reported to function primarily as a transcriptional repressor, however, examples of an activating role in gene expression have also been described (Yuasa et al., 2005; Lugthart et al., 2011). Arsenic trioxide (ATO), an inorganic arsenical, has been investigated as a potential therapy for hematological diseases overexpressing EVI1. Shackelford et al. (2006) found that super-pharmacologic concentrations of ATO degrade EVI1, as well as several fusion proteins containing EVI1, in cell line models of AML. A clinical trial investigating the effect of ATO in combination with thalidomide in myelodysplastic syndrome (MDS), reported hematologic responses in three out of five patients with overexpression of EVI1, including responses in two out of three patients with inv(3)/t(3;3) (Raza et al., 2004). Here, we describe the treatment of a patient with inv(3) AML with the organic arsenical darinaparsin (reviewed in Mann et al., 2009). Although EVI1 expression was not modulated following treatment, we did monitor the response and development of resistance to darinaparsin. By assessing tumor gene expression longitudinally within the patient, we found changes in the alternative NF-κB pathway to be important correlates with response.

## Results

### Medical history and pre-treatment investigations

**Figure 1:**
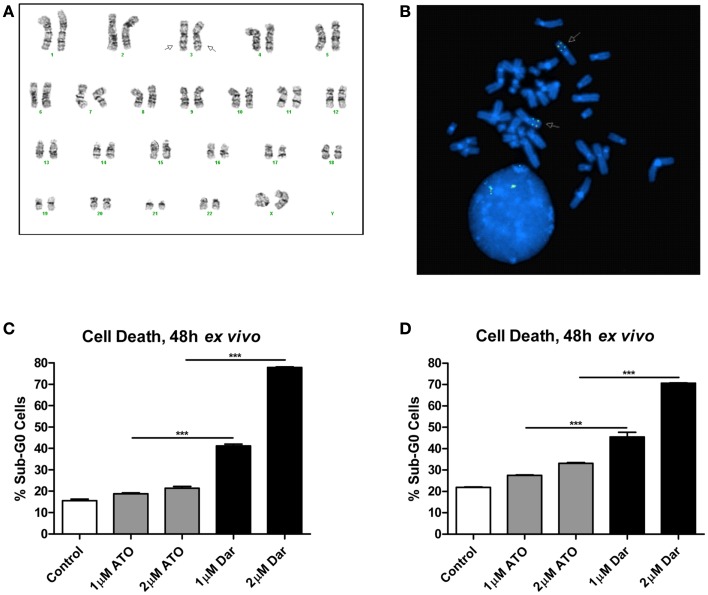
Metaphase karyotype from initial diagnosis **(A)** and fluorescence *in situ* hybridization performed at relapse **(B)** showing biallelic inversion of chromosome 3 (arrows). Also shown are two intact copies of chromosome 7 in this patient **(A)**. Mononuclear cells isolated by Ficoll gradient separation from bone marrow aspirate **(C)** and peripheral blood **(D)** were treated *ex vivo* for 48 h with the indicated doses of arsenic trioxide (ATO) and darinaparsin (Dar) to assess sensitivity to arsenicals. Cell death was measured as the percentage of cells with sub-G_0_ DNA content. Error bars indicate SEM.

The patient was diagnosed with AML in 2005 at the age of 30. The karyotype performed on bone marrow cells at diagnosis was 46,XX,inv(3)(q21q26.2) × 2[21](Figure 1A). She initially underwent two induction regimens followed by an allogeneic stem cell transplant, resulting in a complete remission. After 5 years, the patient’s AML relapsed and she received a total of seven different chemotherapy-containing regimens in an effort to try to control her disease (see Table 1 for details). Fluorescent *in situ* hybridization experiments confirmed the presence of a biallelic inversion of chromosome 3 (Figure 1B). Given previous reports suggesting that malignant cells with inv(3) might be sensitive to ATO, cells from the patient’s bone marrow were collected to determine response to arsenicals *ex vivo* (Raza et al., 2004; Shackelford et al., 2006). As shown in Figure 1C, treatment with therapeutically attainable concentrations of darinaparsin induced significant cell-death, while ATO treatment only resulted in a negligible increase in cell death (Tsimberidou et al., 2009). Of note, ATO (As_2_O_3_) contains twice as much arsenic per mole as darinaparsin (C_12_H_22_AsN_3_O_6_S), thus, 1 μM ATO contains equivalent amounts of arsenic as 2 μM darinaparsin. Given these results, we requested approval from Health Canada to treat the patient with darinaparsin. However, her leukemia progressed rapidly, requiring additional high-dose salvage chemotherapy to control her disease before approval to administer darinaparsin could be obtained. Following the last high-dose regimen, the patient was hospitalized with cytopenia, persistent fever, and poor general condition corresponding to an ECOG performance status of 2. *Ex vivo* treatment of the patient’s peripheral blood mononuclear cells (PBMCs) immediately prior to starting darinaparsin treatment confirmed that tumor cell sensitivity to darinaparsin was intact (Figure 1D).

**Table 1:** **List of regimens used to treat the patient**.

Date	Treatment
April to May 2005	Two induction regimens:
	(1) Cytarabine + daunorubicin (7 + 3)
	(2) Cytarabine + idarubicin (4 + 3)
September 2005	Pre-transplant mobilization:
	Cyclophosphamide + total body irradiation
September 2005	Allogeneic stem cell transplant
August to September 2010	Three high-dose regimens:
	(1) FLAG-Ida (fludarabine, cytarabine, G-CSF, and idarubicin)
	(2) High-dose cytarabine
	(3) Etoposide
November 2010	Investigational treatment:
	Low-dose cytarabine + ribavirin
December 2010	Three salvage chemotherapy regimens:
	(1) Mitoxantrone
	(2) Hydroxyurea
	(3) Etoposide + cyclophosphamide
January to February 2011	Investigational treatment:
	Darinaparsin

### Clinical response to darinaparsin treatment

**Figure 2:**
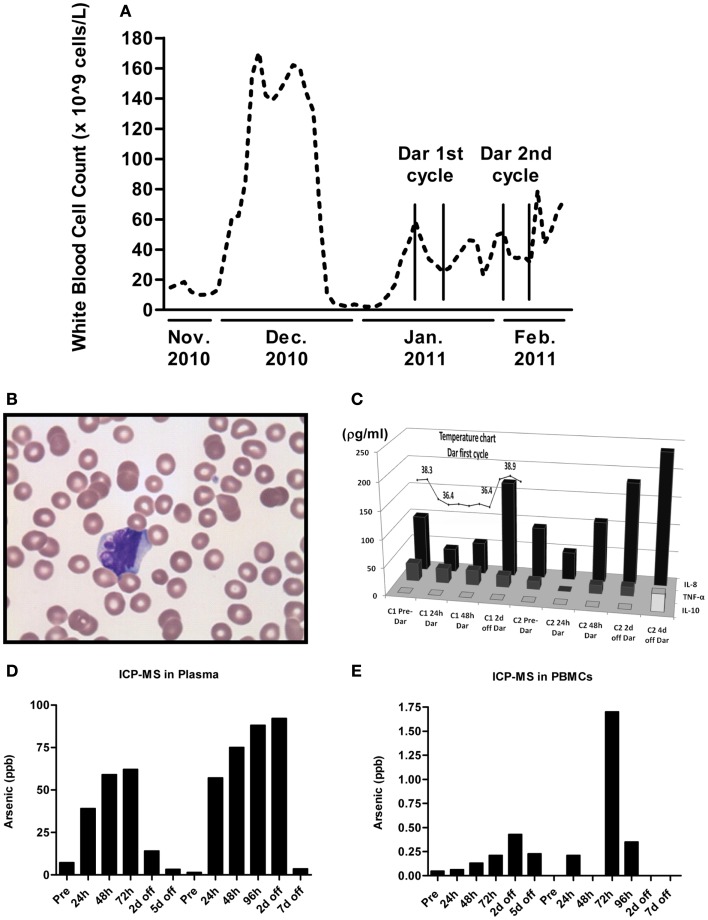
**(A)** White blood cell counts were measured every time the patient was seen at our institution. The beginning and end of cycles of darinaparsin treatment are demarcated with vertical lines. Note that the *x*-axis is not to scale. **(B)** Micrograph of peripheral blood smear stained with Wright’s Giemsa stain while the patient was on darinaparsin treatment. Nuclear blebbing suggests cells are dying by apoptosis. **(C)** While the patient was in hospital, her temperature was monitored daily (measured in degrees Celsius). The temperature curve has been overlaid graphs showing expression of circulating cytokines in plasma, measured by a multiplex immunoassay kit. Only three out of the 11 cytokines measured by the kit were detected at levels above the assay’s limit of detection. IL-8, interleukin 8; IL-10, interleukin 10; TNF-α, tumor necrosis factor-alpha. Samples taken prior to starting treatment with darinaparsin are labeled “Pre” while time points during and after darinaparsin treatment are labeled with cycle number (C1, cycle 1; h, hours; d, days) and time on or off darinaparsin treatment. The patient was allowed to go home 3 days after completing her first course of darinaparsin. Elemental arsenic levels were measured in patient plasma **(D)** and peripheral blood mononuclear cells (PBMCs) **(E)** by inductively coupled plasma mass spectrometry. The highest plasma level of arsenic measured with this dosing schedule was approximately 90 parts per billion (ppb). Given arsenic’s molar mass of 75 g/mol, this is equal to a plasma concentration of 1.2 μM, which corresponds well with the doses used for *ex vivo* experiments in Figures 1C,D.

Peripheral white blood cell (WBC) counts were measured each time the patient was seen at the hospital. As shown in Figure 2A, darinaparsin immediately reduced the patient’s WBC counts and this effect was maintained while the patient remained on drug. The wave-like pattern in WBC counts, with two troughs per treatment cycle, suggests that darinaparsin exerted both immediate anti-cancer effects and more delayed effects. Microscopy of peripheral blood smears, showing nuclear blebbing, suggests cells were dying by apoptosis (Figure 2B). Induction of apoptosis has previously been described as a mechanism of anti-cancer activity of darinaparsin (Matulis et al., 2009).

Within 10 h of the first darinaparsin infusion, the patient’s fever (Figure 2C) and night sweats resolved and she regained her appetite and energy. She was discharged home 3 days after receiving the last dose of the first cycle. She enjoyed a good quality of life, with an ECOG performance status of 1 for more than 30 days, while undergoing her second cycle of darinaparsin as an outpatient. Treatment was well tolerated with no observed side effects. The patient died from extramedullary manifestations and progression of her AML 36 days after receiving the first dose of darinaparsin.

Given the rapid resolution of the patient’s symptoms following darinaparsin administration, we profiled levels of pro-inflammatory cytokines in plasma in response to treatment using a flow cytometry-based multiplex immunoassay kit. While all but three of the 11 cytokines investigated were below the limit of detection of the assay, high levels of interleukin (IL)-8 were detected prior to starting treatment (Figure 2C). IL-8 has both pro-survival and pro-proliferative properties and has previously been shown to be increased in AML patients (Waugh and Wilson, 2008; Kornblau et al., 2010). The patient’s pre-treatment IL-8 levels were about 10 times higher than what has been reported for normal controls (Kornblau et al., 2010). Interestingly, the changes observed in IL-8 plasma levels in response to darinaparsin treatment mirror the changes seen in the patient’s WBC counts (Figure 2A) and temperature (Figure 2C), suggesting that darinaparsin impacts temperature, as well as levels of leukemic cells and inflammatory cytokines (these effects may well be related) and that this may underlie the marked subjective and objective improvement in the patient’s symptoms. Pearson correlation coefficients between IL-8 expression, temperature, and WBC counts suggest that these three variables are indeed strongly positively related (Table 2).

**Table 2:** **Calculation of Pearson correlation coefficients for the relation of temperature, white blood cell counts, and interleukin 8 (IL-8) plasma concentration**.

Date	IL-8 Conc. (pg/mL)	Temp. (Celsius)	WBC counts + 1 day
11 January 2011	99.36	38.3	45.1
12 January 2011	41.69	36.8	33.5
13 January 2011	56.53	36.4	30.3
18 January 2011	170.4	38.6	46.1
Pearson IL-8 vs Temp. Correlation co-eff:		0.883715871	
Pearson IL-8 vs WBC + 1day Correlation co-eff:		0.854039474	
Pearson Temp. vs WBC + 1day Correlation co-eff:		0.998008274	

To determine whether the delayed effects were due to arsenic accumulation in the patient, we measured elemental arsenic levels in patient plasma and PBMCs. Arsenic levels in plasma increased within the first 24 h of treatment and continually rose throughout the treatment period (Figure 2D). After 4–5 days off drug, plasma arsenic levels returned to baseline. Arsenic levels in PBMCs rose slower than plasma levels and remained elevated for several days after discontinuation of darinaparsin (Figure 2E). Thus, while it does not appear that arsenic accumulates in patient plasma under this dosing regimen, it seems that high arsenic levels in PBMCs are maintained several days after discontinuation of darinaparsin.

### Laboratory and correlative investigations

**Figure 3:**
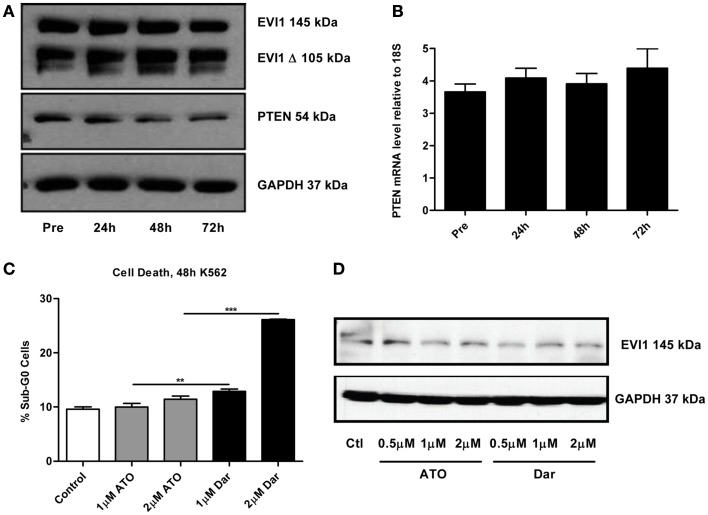
**(A)** Western blot of full-length ecotropic viral integration site 1 (EVI1), a truncated form of EVI1 (EVI1 Δ) and PTEN expression in peripheral blood mononuclear cells at the time points indicated. No fusion of EVI1 and MDS1 was detected in these cells. GAPDH is included as a loading control. **(B)** Levels of PTEN mRNA measured by qPCR. Expression levels are shown as PTEN ΔΔct/GAPDH ΔΔct. Error bars indicate SEM. No statistically significant differences in mRNA levels were found. **(C)** K562 chronic myeloid leukemia cells were treated for 48 h with the indicated doses of arsenic trioxide (ATO) and darinaparsin (Dar) to assess sensitivity to arsenicals. Cell death was measured as the percentage of cells with sub-G_0_ DNA content. Error bars indicate SEM. **(D)** Western blot of EVI1 in K562 cells treated for 24 h with the indicated doses of ATO and darinaparsin (Dar). GAPDH is included as a loading control.

By continuously sampling during the treatment of this patient, we were able to perform correlative studies to investigate potential markers of response and resistance. A defining molecular pathology of inv(3)/t(3;3) AML is the aberrant overexpression of EVI1. Previous studies suggested that arsenic could decrease EVI1 expression, potentially contributing to the cytotoxic effects (Raza et al., 2004; Shackelford et al., 2006). To ascertain the effect of darinaparsin on EVI1, we first analyzed EVI1 protein expression levels in PBMCs by immunoblot. No change in EVI1 protein was observed during the first 72 h of darinaparsin treatment (Figure 3A). After 72 h, there was significant hemolysis of blood samples, preventing reliable quantification at later time-points. We also assessed whether the effect of darinaparsin observed in this patient was due to inhibition of EVI1’s gene regulatory activity by analyzing the expression levels of the tumor suppressor PTEN, a target gene repressed by EVI1, by quantitative PCR and western blot (Yoshimi et al., 2011). No induction of PTEN mRNA or protein was observed during the first 72 h of treatment (Figures 3A,B).

In order to determine the reproducibility of our results obtained in a single patient, we treated the EVI1-expressing myeloid leukemia cell line K562 with ATO and darinaparsin for 24 and 48 hours. Analogous to what was observed in primary leukemic cells (Figures 1C,D), K562 cells were more sensitive to darinaparsin than to ATO treatment (Figure 3C) while neither darinaparsin nor ATO treatment modulated expression of EVI1 protein levels in K562 cells (Figure 3D). Thus, our results do not support modulation of EVI1 expression or repressive activity as part of the anti-leukemic mechanism of darinaparsin in either EVI1-expressing primary cells or cell lines. Note that decreased EVI1 mRNA expression was only described after several months in MDS patients treated with ATO and thalidomide (Raza et al., 2004).

### Potential mechanism of sensitivity/resistance

**Figure 4:**
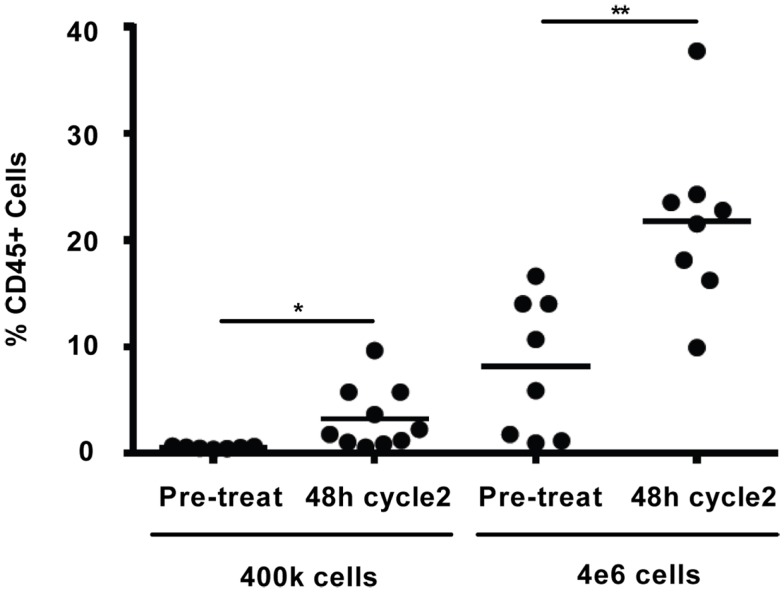
Percentage of engraftment in NOD/SCID/IL2Rγc mice for the indicated cells. Each symbol represents a single animal analyzed 12 weeks after transplantation, mean engraftment is indicated by horizontal bars. The percentage of human CD45 in the BM was determined via flow cytometry.

Comparison of the *in vivo* WBC response to darinaparsin during cycle 1 with the response during cycle 2 suggests the emergence of resistance to darinaparsin (Figure 2A). In particular, the increase in WBC counts after darinaparsin infusion was stopped is greater in cycle 2 than in cycle 1. While the patient was still at home, enjoying a good quality of life at this time, these results suggest that resistance to darinaparsin was starting to develop. Further evidence of a resistant phenotype was provided by xenograft studies in nude mice (Figure 4). In order to measure levels of leukemia initiating cells, we measured the engraftment of patient-derived PBMCs from a pre-treatment blood sample compared to PBMCs isolated during cycle 2. Engraftment is predictive of the number of leukemia initiating cells. Patient PBMCs isolated after 48 h of darinaparsin treatment during cycle 2 showed greater levels of mouse marrow engraftment than pre-treatment PBMCs, suggesting elevated levels of leukemia initiating cells in the post-treatment sample from cycle 2.

**Figure 5:**
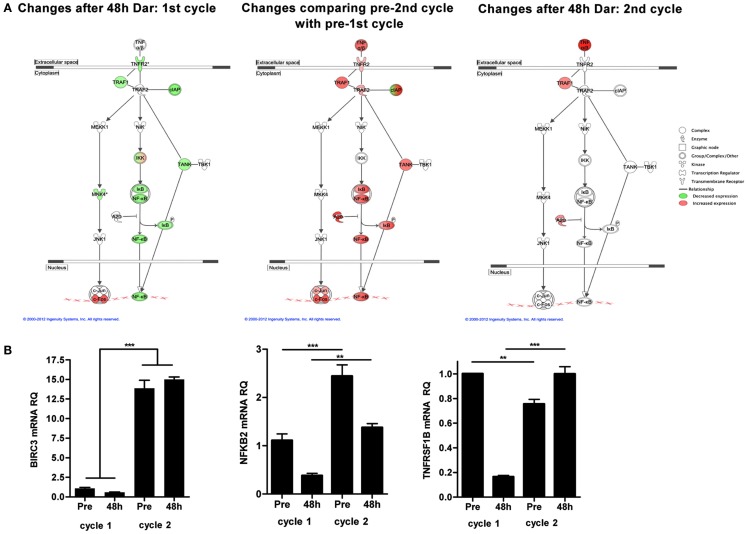
**(A)** Microarray analyses were performed on RNA isolated from peripheral blood mononuclear cells before and 48 h after the start of each cycle of darinaparsin using Agilent Human 60 K expression arrays. The TNFR2/NF-κB alternative signaling pathway was identified as significantly changed (*p* = 3.09 × 10^−3^). Symbols denote gene function (see legend on right). **(B)** Validation of expression of selected genes from the alternative NF-κB pathway (BIRC3, NFKB2, and TNFRSF1B) reveals good agreement with results from microarray analysis. Expression levels are shown as NF-κB pathway ΔΔct/GAPDH ΔΔct. Error bars indicate SEM. BIRC3, baculoviral IAP repeat containing 3; NFKB2, nuclear factor of kappa light polypeptide gene enhancer in B-cells 2; TNFRSF1B, tumor necrosis factor receptor superfamily, member 1B.

To explore potential mechanisms of resistance to darinaparsin, we performed cDNA microarray analyses on PBMCs prior to and 48 h after the start of each cycle of darinaparsin. The alternative NF-κB pathway was identified as one of the most modulated pathways during the course of darinaparsin treatment using Ingenuity Systems Pathway Analysis (Figure 5A, left panel). Expression patterns of three genes in the alternative NF-κB pathway were validated by quantitative PCR using gene-specific primers as shown in Figure 5B. There is good agreement between results obtained by microarray and quantitative PCR. Darinaparsin decreased expression of several members of the alternative NF-κB pathway after 48 h of treatment. This correlates with the observed decrease in production of IL-8 (Figure 2C), a well-characterized NF-κB target gene. Furthermore, gene expression in PBMCs prior to the second cycle of darinaparsin revealed an upregulated alternative NF-κB pathway that was not inhibited by darinaparsin during the second cycle, perhaps foreshadowing the emergence of resistance (Figure 5A, center and right panels). The attenuation of the response of the alternative NF-κB pathway to darinaparsin treatment coincides with emergence of resistance, thereby strengthening the hypothesis that this pathway plays a role in the sensitivity/resistance of inv(3) AML cells to darinaparsin.

## Discussion

Herein, we describe for the first time the treatment of a patient with inv(3) AML with the organic arsenical darinaparsin. Darinaparsin stabilized disease in a heavily pretreated patient and greatly improved quality of life. Therapy was well tolerated with almost immediate clinical benefit and no observed side effects. Clinical benefit was suggested by a simple *ex vivo* cell-death assay performed prior to starting therapy, indicating that this may be useful as a predictive marker of *in vivo* response. Unexpectedly, we did not find evidence that treatment directly affected EVI1 levels or activity. Instead, our results indicate that inhibition of the alternative NF-κB pathway by darinaparsin correlated with response and plasma levels of the inflammatory cytokine IL-8.

The strategy outlined here serves as an example of the power of a personalized medicine approach in patients for whom no accepted standard therapy is available. In particular, for patients suffering from uncommon types of malignancies, it is imperative to gather as much information on treatment effect, response, and resistance at every given opportunity. This series of experiments highlights how multiple platforms can be used to assess mechanisms of response and resistance. Here, we included everything from genomics to *in vivo* stem cell analysis.

In this specific case, the literature suggested that arsenicals might be efficacious in malignancies with inv(3), thus we tested two arsenicals using a simple *ex vivo* death assay. Interestingly, the drug that performed the best in our *ex vivo* assay was not ATO [the drug for which activity in inv(3) malignancies had previously been reported], but rather the organic arsenical darinaparsin (Raza et al., 2004; Shackelford et al., 2006; Mann et al., 2009). Darinaparsin has previously been tested in hematological malignancies, including acute leukemias (Clinical trial ID: NCT00592046). Encouragingly, the patient experienced marked subjective and objective improvement after starting darinaparsin, however, after about a month, her condition rapidly deteriorated. Our results highlight a key advantage of longitudinal sampling of the same patient over his or her entire disease trajectory. The availability of blood samples from both the time during which the malignant cells exhibited sensitivity to treatment (cycle 1) and the time at which resistance was starting to become apparent (cycle 2) allowed us to compare treatment responses at these time points. Using an unbiased gene expression based approach, we identified the alternative NF-κB pathway as being constitutively activated in the patient’s AML blasts prior to treatment and showed that this activation was attenuated after 48 h of darinaparsin. However, prior to the start of cycle 2, the NF-κB pathway was upregulated further and was no longer responsive to darinaparsin treatment (Figure 5). The abrogation of a putative mechanism of response at the time of emergence of resistance strengthens the hypothesis that this pathway plays an important role in resistance/sensitivity to darinaparsin in this patient. Levels of circulating IL-8 in plasma may function as a surrogate marker of NF-κB pathway activation and could conceivably be used to monitor treatment response in IL-8 secreting AML.

Based on the results outlined above, further investigation is warranted into (1) EVI1 overexpressing AMLs and (2) AMLs with activation of the alternative NF-κB pathway as potential targets for darinaparsin treatment.

## Materials and Methods

All clinical investigations and treatments were performed with patient consent under a compassionate use protocol approved by the Jewish General Hospital Research Ethics Committee and Health Canada.

### Cells

K562 cells were purchased from ATCC and grown in IMDM medium supplemented with 10% fetal bovine serum and antibiotics.

### Standard cytogenetics and FISH

G-banded karyotypes were obtained from bone marrow mononuclear cells according to standard cytogenetic procedures and described according to the International System for Human Cytogenetic Nomenclature 2009. FISH experiments were performed on interphasic nuclei and metaphases using the EVI1 Breakapart probe LPH036 (Cytocell, Cambridge, UK).

### Sample preparation

Peripheral blood was collected in heparin tubes prior to starting treatment and every time the patient was seen in hospital. The blood was spun down to remove the plasma (stored at −80°C) followed by isolation of PBMCs by Ficoll gradient separation. RNA, DNA, and whole cell extracts were isolated as described below. Surplus PBMCs were frozen in 10% DMSO/90% fetal bovine serum at −80°C.

### Propidium Iodide (PI) stain of *ex vivo* treated bone marrow cells and PBMCs

For a detailed description see Hardin et al. (1992). Briefly, cells were washed once in FACS wash buffer (5 mM NaN_3_ in PBS supplemented with 5% fetal bovine serum) and then stained with 50 μg/mL PI (Sigma) in hypotonic buffer (0.1% sodium citrate and 0.1% Triton X-100 in ddH_2_0). Approximately 500,000 cells/condition were stained with 0.5 mL PI stain and analyzed on a BD Biosciences FACSCalibur flow cytometer. Cell death was measured as the percentage of cells with sub-G_0_ DNA content.

### ICP-MS

Elemental arsenic levels were measured in patient plasma and PBMCs by inductively coupled plasma mass spectrometry (Chemical Solutions Ltd, Mechanicsburg, PA, USA).

### Immunoblotting

Whole cell extracts were made from patient samples and K562 cells using Tris/NaCl/triton x-100 buffer (50 mM Tris-HCl pH 8.0, 150 mM NaCl and 1% triton X-100 supplemented with protease inhibitor cocktail (Roche) and PhosStop (Roche)(. Proteins were separated by SDS-PAGE and transferred to nitrocellulose membrane. Primary antibodies for PTEN, EVI1, and GAPDH were all purchased from Cell Signaling. Specific binding was detected with horseradish peroxidase-labeled secondary antibodies and visualized with enhanced chemiluminescence.

### qPCR

RNA was extracted from patient samples using Qiagen AllPrep DNA/RNA mini kit. RNA was converted to cDNA using Superscript II reverse transcriptase (Invitrogen). Specific qPCR amplification was performed using a 7500 Fast Thermocycler (Applied Biosystems) and either Taqman chemistry (PTEN, 18S, and GAPDH) or SYBR green chemistry and specific primers. BIRC3: 5′-TCC GTC AAG TTC AAG CCA GTT-3′ and 5′-GGG CTG TCT GAT GTG GAT AGC-3′; TNFSF1B: 5′-GGC CAG ACC AGG AAC TGA AA-3′ and 5′-GAT GAA GTC GTG TTG GAG AAC GT-3′; and NFkB2: 5′-ACG AGG GAC CAG CCA AGA T-3′ and 5′-GCA CGA GGT GGG TCA CTG T-3′.

### Circulating cytokine determination

Cytokine profiling of patient plasma samples was done using a Human Th1/Th2 11plex FlowCytomix Multiplex kit (eBioscience, San Diego, CA, USA) according to the manufacturer’s instructions.

### Mouse work

Studies were performed with approval from the University of Rochester Institutional Review Board, IUCOC. NOD/SCID/IL2Rγc mice were sublethally irradiated with 2.7 Gy (270 rad) using a RadSource X-ray irradiator (RadSource, Boca Raton, FL, USA) the day before transplantation. Cells to be assayed were thawed, counted, and then injected via tail vein in a final volume of 0.2 mL of PBS with 0.5% FBS. After 12 weeks, animals were sacrificed and BM was collected. To determine human cell engraftment the BM cells were labeled with antihuman CD45 (Becton Dickinson) and analyzed via flow cytometry.

### Gene expression profiling

Microarray analyses were performed using Agilent (Mississauga, ON, USA) Human 60 K expression arrays at Genome Quebec. Data were analyzed using FlexArray and Ingenuity Pathway Analysis Software.

Raw text files for the one-color Agilent array were imported in R version 2.14.0 using the package limma (PMID 16646809). We processed the data by first subtracting the background using normexp, took the log2 of the remaining intensity and applied quantile normalization to the entire set of arrays. Differential expression of genes was computed using limma (PMID 16646809). We considered genes as differentially expressed if the mean normalized signal of the two conditions is higher than five and the differential expression is greater than twofold change.

### Patient treatment protocol

Darinaparsin (300 mg/m^2^) was administered by intravenous infusion on five consecutive days followed by 16 days off drug. Following the end of the first 21-day cycle, this protocol was repeated for cycle 2.

### Statistics

For *in vitro* experiments, all error-bars represent the standard error of the mean of three replicates. Significance was determined by one-way analysis of variance followed by Newman–Keuls post-tests using Prism version 3.0 (GraphPad software, San Diego, CA, USA). For the mouse work, significance was calculated using an unpaired *t*-test (two-tailed).

*, *p* < 0.05; **, *p* < 0.01; ***, *p* < 0.001.

Pearson correlations in Table 2 were calculated using the correlation function in Microsoft Excel with the data points shown.

## Conflict of Interest Statement

Robert A. Morgan is Senior Vice President, Regulatory Affairs and Quality/Pharmaceutical Development of ZIOPHARM Oncology, Inc. and a stockholder of ZIOPHARM. ZIOPHARM is the licensee of darinaparsin and is developing darinaparsin for various indications in oncology.
